# Parenting Profiles in Military Families: Intervention-Related Transitions and Relationships to Child Adjustment

**DOI:** 10.1007/s11121-024-01721-7

**Published:** 2024-09-16

**Authors:** Sun-Kyung Lee, Abigail H. Gewirtz, Timothy F. Piehler

**Affiliations:** 1 Department of Psychology, Arizona State University, 950 S. McAllister Ave, TempeTempe, AZ 85287-1104 USA; 2 Department of Family Social Science, University of Minnesota, St. Paul, MN USA; 3 Paul Baerwald School of Social Work, Hebrew University of Jerusalem, Jerusalem, Israel

**Keywords:** Latent transition analysis, Post-deployed military families, Child maladjustment, Evidence-based program

## Abstract

Parenting programs aim to improve parenting quality, which may, in turn, support various aspects of child development, including behavior and mental health. However, parenting interventions show considerable heterogeneity in response patterns across different families, demonstrating that they are not one-size-fits-all programs. This variability points to a need for greater understanding of which families benefit most from these interventions and how to improve response among those who do not. Following this literature gap, this study employed a person-centered approach to identify different parenting profiles associated with heterogeneity in treatment responses to a family-based prevention interventions adapted for military families. This study used data from a randomized controlled trial of the ADAPT intervention for 336 US military families in which at least one parent had deployed to war. Latent profile analyses revealed three unobserved parenting profiles among mothers and fathers, reflecting *High positive*, *Moderate positive*, and *Coercive* parenting styles. Latent transition analysis (LTA) suggested that the ADAPT program led to improvements in parenting, particularly among mothers who began the program with moderate or typical levels of parenting skills, and that these positive changes in parenting may help to decrease child externalizing problems. For fathers, the ADAPT program was most effective in preventing declines in positive parenting among those with initially typical levels of positive parenting. Overall, study findings demonstrate considerable heterogeneity in parenting behavior among a sample of military families that is associated with variability in parent responses to the evidence-based parenting program.

**Supplementary Information:**

The online version contains supplementary material available at 10.1007/s11121-024-01721-7.

Parenting programs aim to improve parenting quality, such as reducing harsh parenting and increasing use of positive support for appropriate behavior, which may, in turn, support children’s healthy development and behavior (Epstein et al., 2015). Interventions targeting parenting are complex as parenting consists of values, knowledge, and skills and is influenced by multiple factors in the parent(s)’ environment (Belsky, 1984). Many studies have demonstrated the overall efficacy of parenting interventions (e.g., Triple P, Incredible Years) to improve parenting skills and child adjustment for families of diverse backgrounds (Barlow & Stewart-Brown, 2000; Gross et al., 2003; Hutchings et al., 2007; Kim et al., 2008; Sanders et al., 2000; Scott et al., 2010). However, parenting interventions demonstrate considerable variability in their effectiveness with some families benefiting and others not (de Graaf et al., 2008; Menting et al., 2013; Reyno & McGrath, 2006; Thomas & Zimmer-Gembeck, 2007; van Aar et al., 2019). Surprisingly, in the parenting intervention literature, little consideration has been given to which parenting dimensions (e.g., limit setting, praise) are most crucial to intervention programming. We also know little about how parents presenting with different profiles of parenting skills may differentially respond to programming.

## Strengthening Military Families

Military families, including those in the National Guard and Reserves, often experience multiple stressors such as prolonged separations and extensive changes, with limited access to parenting support and resources (Mmari et al., 2009). Exposure to traumatic events increases the risk of posttraumatic stress disorder (PTSD), depression, and substance use (Asnaani et al., 2014; Jacobson et al., 2008; Polusny et al., 2011). Reintegration, often considered the most stressful period in the deployment cycle, demands that service members return to their family roles like reestablishing parent-child relationship, while coping with the challenges resulting from combat, such as physical injuries, PTSD, and other psychiatric illnesses (Gewirtz et al., 2010; Julian et al., 2018). Civilian family members may also face challenges due to deployment, including disrupted family routines, mental health problems, reintegration after extended separations, impaired parenting practices, and elevated risk for child maltreatment (Creech et al., 2014; Mansfield et al., 2010; Paley et al., 2013). Children with military parents suffering from severe mental health problems are negatively affected, showing increased risk for anxiety and adjustment problems (Giff et al., 2019; Gewirtz et al., 2018a; Lester et al., 2016; Gorman et al., 2010).

National Guard soldiers who recently returned from deployment showed a preference for family-based interventions over individual treatments to address post-deployment mental health and stress in child-rearing practices (Khaylis et al., 2011). Without effective interventions for military families, their traumatic stress may increase the risk of divorce, child maladjustment, and negatively impact the well-being of service members. Evidence-based parenting programs has been demonstrated to improve parenting and promote positive child adjustment in at-risk families such as those who have experienced military deployment (Forgatch & Patterson, 2010). After Deployment, Adaptive Parenting Tools (ADAPT) is the first evidence-based parenting intervention designed primarily for military families with school-aged children (age 4–12). It is rated as a promising program in The Clearinghouse Continuum of Evidence (2023). The program was adapted from the empirically validated parenting program known as the Generation Parent Management Training: Oregon Model (GenerationPMTO; previously known as PMTO; Forgatch & Patterson, 2010). ADAPT has been demonstrated to improve parenting, children’s emotional, behavioral and social adjustment, and parental mental health outcomes (Gewirtz et al., 2018b; Zhang et al., 2020; DeGarmo & Gewirtz, 2018).

## Heterogeneity in Parent Training Program Responses

One major goal in the field of prevention science is to better understand which families are likely to benefit the most from preventive intervention programming (Fairchild & Mackinnon, 2014). Even when receiving the same parenting program, some parents are likely to benefit while others are not (van Aar et al., 2019). Parenting programs may produce heterogeneous effects due to various factors: intrapersonal (e.g., demographics), interpersonal (e.g., partner relationship), community (e.g., access to external support), and cultural (e.g., values) factors (Leijten et al., 2013; Wymbs, 2011; Byrnes & Miller, 2012; Niec et al., 2015; Weeland et al., 2021). van Aar and colleagues (2019) demonstrated that about one-third of families showed a limited or nonresponse to a parent-focused intervention. The effects of ADAPT on parenting practices also have shown to be influenced by parents' initial PTSD symptoms and emotional avoidance. For example, fathers with clinical levels of PTSD at baseline were found to be less effective (Chesmore et al., 2018), mothers with higher experiential avoidance exhibited more supportive emotion socialization after the intervention (Zhang et al., 2018), and fathers with emotional difficulties showed greater improvements in emotion regulation (Zhang et al., 2023). These findings highlight how diversity in parents’ initial parenting competencies is related to variability in intervention response.

Despite variability in parent response, parenting programs tend to be delivered in a one-size-fits-all approach, meaning all parents receive the same parent training. It is critical to better understand how unique parenting profiles may relate to unique patterns of response to the intervention. If researchers can understand the unique characteristics of those who benefit or do not benefit from specific approaches, programming may be tailored to best meet individual needs and produce stronger effects (Turney, 2015).

A barrier to intervention tailoring has been a lack of consistent understanding regarding who is most or least likely to benefit from parenting programs (Forgatch & Patterson, 2010; Thomas & Zimmer-Gembeck, 2007). This limited understanding may in part result from common statistical approaches to investigating trial outcomes obscuring heterogeneity in response. Most studies assume homogeneity of families within a sample when examining outcomes (Pelham et al., 2017) and model only linear relationships between family characteristics and outcomes when examining variability in intervention effects (Leijten et al., 2013). Moreover, much of the research investigating variability in response has focused on single variable moderators rather than a broader selection of characteristics or behaviors likely to influence response. Yet, the majority of prior studies still focus on mothers or samples that consist primarily of mothers, and there is a lack of understanding regarding fathers’ responses to parenting programs (Tiano & McNeil, 2005; Fletcher et al., 2011; Panter-Brick et al., 2014).

Pre-intervention parenting skills in particular represent a promising set of characteristics that seem likely to be related to response to programming targeting these skills (van Aar et al., 2019). To the best of our knowledge, there is limited existing research investigating how broader parenting profiles encompassing a variety of parenting skills may change in response to a parenting program and how pre-intervention parenting profiles may predict variable intervention response (Piehler et al., 2022; van Aar et al., 2019).

## Use of Latent Transition Analysis to Understand Variability in Intervention Response

The use of a longitudinal person-centered approach such as latent transition analysis (LTA) may address some of the limitations of previous work investigating variability in response to parenting programming. LTA allows for the identification of unobserved within-sample subgroups and changes in those subgroups over time. LTA is a longitudinal extension of the latent class mixture model which is used to model changes over time in membership in categorical variables. It is a well-suited method to explore changes in group membership (i.e., unobserved latent nominal classification) over time while accounting for the measurement error and the uncertainty of group membership (Nylund et al., 2007). This method can also include predictors and distal outcomes to extend the understanding of the transitional process (McGrath & Tschan, 2004; Nylund et al., 2007). By incorporating an intervention effect and distal outcomes, LTA can answer whether the intervention effect varies across classes over time, and how distal outcomes are predicted by different transitions between classes.

An LTA approach has been used in other intervention contexts to better disentangle how different profiles may be associated with variability in response to an intervention. Connell and colleagues (2008) investigated the effectiveness of the family check-up intervention, a brief, family-focused, motivationally-based preventive intervention, using LTA. They found that youth in a comorbid group experiencing high internalizing and externalizing symptoms were more likely to transition into a normative group (i.e., low likelihood of problems) when receiving the intervention. Several studies have incorporated LTA to evaluate how interventions may differentially impact certain subgroups (Mackesy-Amiti et al., 2013; Roberts & Ward, 2011). However, those studies did not explore how those qualitative nominal changes within groups may be related to key distal outcomes.

While LTA has been used less frequently in prevention science and family-focused studies, person-centered approaches are increasingly being used to evaluate variability in intervention effects or behavior change (Collins & Wugalter, 1992). For example, Weeland et al. (2023) similarly used latent profiles to assess how baseline parenting profiles predicted intervention outcomes, and Pelham et al. (2017) applied profiles to identify which families benefited most from a brief family-focused intervention. The use of person-centered methods within prevention may lead to the development of more effective interventions for different subgroups.

## The Current Study

Parenting interventions show considerable heterogeneity in response patterns across different families, demonstrating that they are not one-size-fits-all programs. However, there is a lack of understanding of who benefits from parenting interventions and how to increase benefits and program efficiency. Existing studies have focused on single moderators to understand the variability in intervention-related change rather than looking more broadly at profiles of multiple variables. Applying a longitudinal person-centered analysis such as LTA provides additional understanding of this variability beyond the advantages of traditional latent growth modeling studies by examining how broader profiles of parenting behaviors may be related to change or stability in those profiles following an intervention relative to control participants. Therefore, this study investigated the change in parenting profiles of parents in military families after participating in the ADAPT parenting intervention. Specific research questions were as follows:

1. Do mothers and fathers in military families significantly change their parenting profile group after participating in the evidence-based ADAPT parent training program relative to control participants?
2. Are certain pre-intervention parenting profiles more likely to be associated with change to more adaptive post-intervention parenting profiles?
3. Are changes in parenting profiles over time associated with distal child externalizing and internalizing behavior outcomes?

## Method

### Participants

The sample included 336 (294 fathers, 314 mothers, and 336 children) National Guard and Reserve families. Families were eligible for participation in the study if they had at least one child living with them (4- to 13-year-old) and at least one parent who had been deployed to recent conflicts in Iraq and/or Afghanistan. Of the 336 families, most had a deployed father and a nondeployed mother (87.41%). The average length of marriage with current partners was 9.6 years (*SD* = 5.3). The fathers were, on average, 37.47 years old (*SD* = 6.43; range 23–58), predominately Caucasian (89.62%); a small percentage were African American (5.00%), Asian American (2.31%), Pacific Islander (0.38%), and multiracial (2.69%). The mothers were on average 35.72 years old (*SD* = 5.84; range 23–51), predominately Caucasian (95.06%), with a small percentage identifying as African American (1.90%), Asian American (1.14%), Pacific Islander (0.38%), Native American (0.38%), and multiracial (1.14%). Most families were middle class (41.5% of families reported annual household income between $40,000 and $79,999 and 30.7% between $80,000 and $119,999). Focus children (55.5% girls) were on average 8.41 years old (SD = 2.52) at baseline. These sample characteristics are representative of the NG/R parent population with school-age children in the Midwest.

### Procedure

Participants were recruited through multiple means such as presentations at military-sponsored events, outreach at military organizations, media, mailing, flyers, and word-of-mouth. Interested parents completed an online screener and consented to participate in the study if they were eligible. After completing an online survey and in-home assessment at baseline, the families were randomly assigned to the intervention or control group in a 6:4 ratio. The intervention group was involved in 14 group-based parent training sessions while the control group received services as usual (a print or online list of parenting resources). The data were collected at baseline (T1) and three follow-up assessments: 6-month (T2), 1-year (T3), and 2-year (T4). The current study focused on the (annual) T1, T3, and T4 assessments, in which observed parenting behaviors were recorded. Parents received $25 for completing the online assessment, and each family was given $50 for completing the in-home assessment. Children were also given small gifts, valued at around $5, for their participation in the in-home assessment. All procedures adhered to ethical guidelines and were approved by the Institutional Review Board at the University of Minnesota (IRB number: 1005S82692).

### ADAPT Intervention

After Deployment, Adaptive Parenting Tools (ADAPT) is a parent-focused preventive intervention program, and was delivered, in this study, as a 14-week face-to-face group-based program.^1^ ADAPT targets teaching of six core effective parenting skills, five of which are identified in GenerationPMTO: (1) skill encouragement, (2) effective problem-solving, (3) warmth and positive involvement, (4) monitoring, and (5) providing effective discipline (Forgatch & Patterson, 2010; Patterson, 2005). A sixth core parenting skill, emotion coaching, was added in ADAPT along with tailored materials specific to military families and the deployment context. More details on the ADAPT curriculum and key content can be found in Gewirtz et al., (2014). Each 2-h group consists of 6–15 parents with 2–3 certified trained facilitators, who were either NG/R military members or military or non-military providers (e.g., social workers, school counselors). During weekly sessions, parents learn parenting skills through observation, role play, and discussions, and are given access to a website with supplemental resources. The ADAPT groups were held in community locations (e.g., churches, libraries, or community colleges) within the Minneapolis/St. Paul metropolitan area and typically within 40 min driving of family homes. To ensure implementation fidelity, group facilitators underwent extensive training and received ongoing supervision, with regular fidelity checks using the Fidelity of Implementation System (FIMP; Knutson et al., 2019) (originally developed for GenerationPMTO) conducted through session observations. ADAPT has been demonstrated to improve parenting and child outcomes (Gewirtz et al., 2018b).

### Measures

#### Observed Parenting: Family Interaction Tasks (FITs)

Parenting practices were directly observed from parent-child interactions during structured FITs. Total FITs assessment times ranged between 40 and 60 min and included a series of activities focused on problem solving, discussing deployment-related concerns, planning a fun family activity, teaching games, and monitoring. 

Five parenting practices have been previously investigated by the social interaction learning (SIL) model: (1) *problem-solving* (e.g., quality of parent-child solution; 9 items (1 = untrue to 5 = very true); *α* = 0.87–0.89; ICC = 0.88–0.94), (2) *skill encouragement* (e.g., promoting children’s skill development through encouragement; 8 items (1 = untrue to 5 = very true); *α* = 0.76–0.83; ICC = 0.72–0.76), (3) *monitoring* (e.g., knowledge of their child’s daily activities; 4 items (1 = untrue to 5 = very true); *α* = 0.60–0.71; ICC = 0.74–0.64), (4) *positive involvement* (e.g., parent’s warmth; 10 items (1 = never to 6 = always); *α* = 0.75–0.76; ICC = 0.76–0.84), and (5) *harsh discipline* (e.g., overly strict, inconsistent; 8 items (1 = never to 6 = always); *α* = 0.75; ICC = 0.58–0.78). Blinded, trained coders scored each parenting practice observed in the FITs using the Coder Impressions System (Forgatch et al., 1992). Prior studies demonstrate good construct validity and high inter-coder reliabilities of these observed parenting practices (Forgatch & DeGarmo, 1999). For both mothers and fathers separately, items were averaged to create composite scores for each parenting indicators. The T1 and T3 FITs data were used for person-centered analysis.

#### Child Adjustment Outcomes: BASC-2 PRS

Child internalizing and externalizing behaviors were measured by parents’ reports on the Behavioral Assessment Scale for Children – Parent Rating Scale (BASC-2 PRS; Reynolds & Kamphaus, 2004). The BASC-2 is a widely used measure of child emotional and behavioral functioning with high internal consistency and test-retest reliability. Internalizing symptoms include depression, anxiety, and somatization subscales. Externalizing behavior includes hyperactivity, aggression, and conduct problems subscales. All items are rated on a 4-point scale regarding the frequency of the child’s behavior (0 = never to 3 = almost always). The study used BASC-2 T-scores at T1 and T4, and the average of both mother and father reports on child outcome measures to examine distal outcomes.

#### Intervention Status

The intervention status was coded as 1 (*Intervention*) and 0 (*Control*). This study utilized an intent-to-treat analysis.

#### Covariates

Marital status (1 = *married*, 0 = *not married*) and baseline (i.e., T1) assessments of the child outcome were included as a covariate.

### Analyses

This study employed a latent transition analysis (LTA), an extension of latent profile analysis (LPA) that uses longitudinal data to identify how subgroup status changes over time. LTA is an appropriate analysis to examine qualitatively distinct behavioral patterns across time points. Using separate models for mothers and fathers, this study followed Nylund’s (2007) 5-step procedure using M*plus* version 8.0.

In step 1, the latent class measurement models for both parents at two time points (T1 & T3; total four models) were explored separately. Each LPA model included 5 indicators of parenting practices (i.e., problem-solving, skill encouragement, monitoring, positive involvement, and harsh discipline). The best fitting number of classes was indicated by lower values of the Akaike information criterion (AIC; Akaike et al., 1987), Bayesian information criterion (BIC; Schwarz, 1978), and adjusted BIC (aBIC; Sclove, 1987); significant difference in the adjusted Lo-Mendell-Rubin likelihood ratio test (adjusted LMR-LRT; Lo et al., 2001). Also, the proportion of the sample within each class and profile interpretability was considered in order to obtain quantitatively and qualitatively meaningful profiles during class enumeration.

In step 2, a cross-sectional transition of profiles across time provided preliminary indication of class transition, and measurement invariance was examined using the chi-square difference test. Then in step 3, the specification of LTA was explored without the intervention effect. Accounting for classification uncertainty, the auto-regressive path was added in the model to predict the transition from T1 to T3 latent profiles. This step provided the transition probabilities (Collins & Lanza, 2009).

In step 4, the ADAPT effect was added in the LTA model. This examined whether participation in ADAPT predicted class membership at T3. This step looked at the changes in the transition probabilities for parents involved in the ADAPT program versus the control condition. Finally, in step 5, distal child outcomes (externalizing and internalizing behaviors) were added to the step 4 model. This step examined how profile transition patterns predicted child adjustment at 24-month follow-up (T4). The mean differences in child adjustment variables of latent transition groups were tested by applying the Wald test.

#### Missing Data

Little’s MCAR test was conducted on all measures that were included in the analysis. The percentage of missing data in fathers’ variables ranged from 2.4 to 26.4%, and in mothers’ variables ranged from 2.3 to 25.4%. The test showed that the pattern of missing values was not completely random among father study variables, *χ*^2^ (174) = 230.551, *p* < 0.01, and mother study variables, *χ*^2^ (180) = 217.462, *p* < 0.05. The missingness correlated with participant attrition over the study with about 25% attrition after 1 year. Full information maximum likelihood (FIML) was used to address missing data in the analysis.

## Results

Descriptive statistics for both fathers and mothers are shown in Supplementary Table 1. There was not a significant association between intervention status and other baseline variables, supporting successful randomization.

**Fig. 1:**
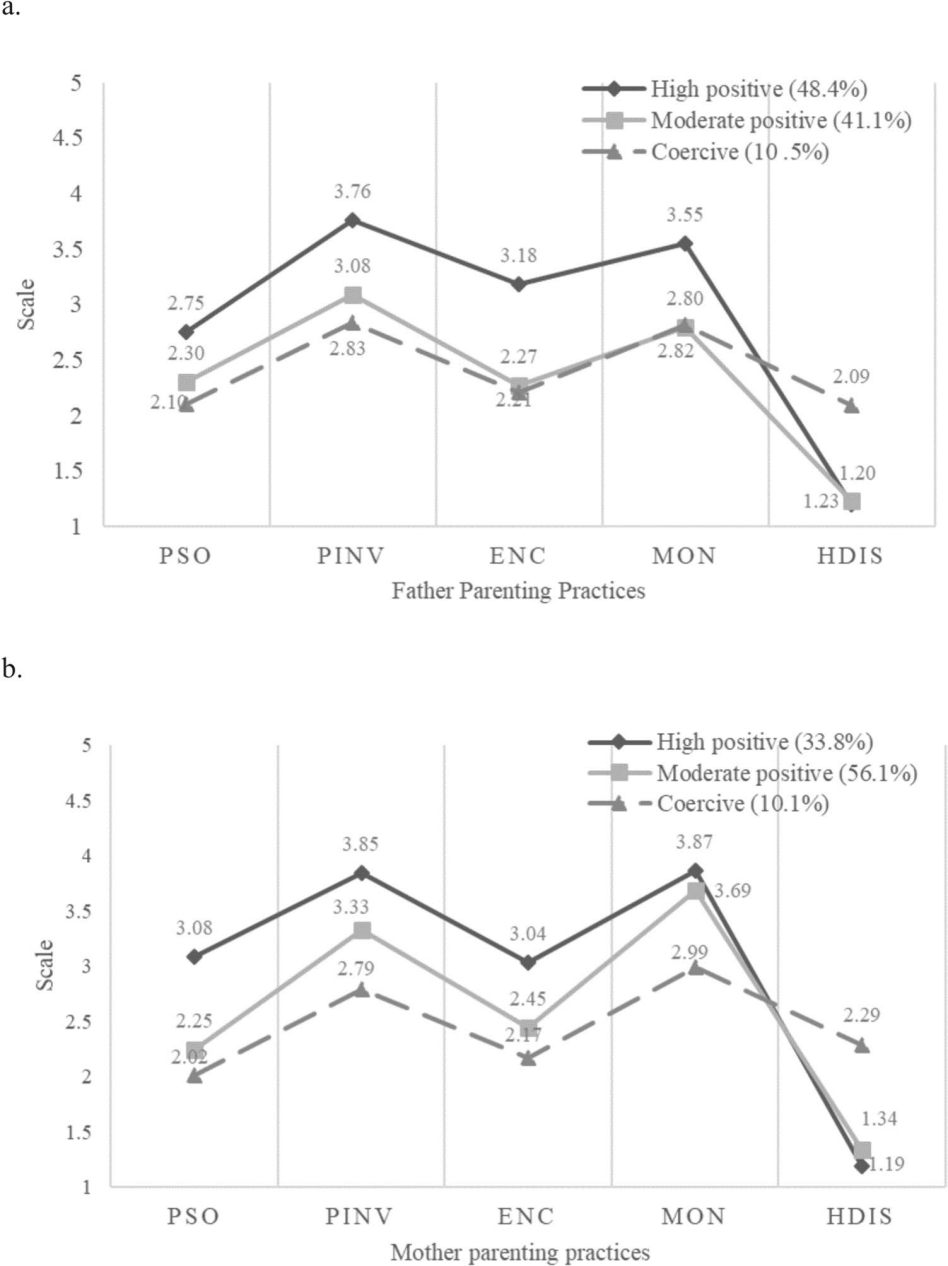
Fathers’ and mothers’ parenting profiles. *Note.* PSO, problem solving; PINV, positive involvement; ENC, encouragement; MON, monitoring; HDIS, harsh discipline

First, separate LCAs at baseline and 1-year follow-up (T3) were examined for both mothers and fathers to determine the optimal number of classes at each time point. After balancing fit indices, parsimony, and interpretability, 3-class models were selected for mothers and fathers across both time points to show parenting transitions (refer to Supplementary Table 2). While 4-class models showed minor improvements on fit indices, these models each included a class with less than 20 parents. These small classes did not represent a meaningful addition to the 3-class models and may represent overextraction of classes in the 4-class model. Figure 1 illustrates the mean score for each of the parenting skill on latent profile membership for both parents. The latent profiles showed overall consistency across parenting skills that the profiles were labeled as the *High positive*, *Moderate positive*, and *Coercive* parenting groups based on their level of positive parenting skills and harsh discipline score. *Coercive* parenting groups in both parents showed distinct scores in harsh discipline compared to the other profile groups. For fathers, there was lower variability in their encouragement and monitoring skills across classes when compared to the mothers.

Next, the measurement invariance test showed that profiles at baseline were significantly different to the T3 profiles. However, due to the primary focus on class transitions, the latent profiles were set as invariant across time points to allow for examination of the movement between corresponding profiles at each time point rather than examining the movement into different profiles caused by the change in the profile definitions at the follow-up assessment. Using corresponding classes at T3, the cross-tabulation of membership suggested shifts in the proportions of profile membership for both parents, indicating change in parenting behavior over time (Supplementary Table 3).

In Step 3, the transition probabilities without the intervention effect was consistent with changes in parenting behavior over time (Table 1). For both mothers and fathers, the transition probability values in the bottom-left diagonal (i.e., percentage of people who moved toward more positive parenting) were higher than the values of the top-right diagonal (i.e., percentage of people who moved toward low positive parenting). Both mothers and fathers showed high stability over time when they belonged in the *High positive* group at baseline (fathers = 88.4%; mothers = 86.9%).

**Table 1:** Transition probabilities

**Fathers**	1-year follow-up (T3)		
Baseline (T1)	*High positive*	*Moderate positive*	*Coercive*
*High positive*	**0.88**	0.12	0.00
*Moderate positive*	0.50	**0.41**	0.10
*Coercive*	0.29	0.62	**0.09**

**Mothers**	1-year follow-up (T3)		
Baseline (T1)	*High positive*	*Moderate positive*	*Coercive*
*High positive*	**0.87**	0.13	0.00
*Moderate positive*	0.42	**0.52**	0.06
*Coercive*	0.31	0.58	**0.11**

Stable transitions are bolded

In Step 4, the inclusion of intervention status allowed for a comparison of the rates of transition across the intervention groups. Latent transitional probabilities in the intervention and control groups as well as the difference between the two are shown in Table 2. Overall, the differences between the intervention and control transition probabilities were larger for mothers than they were for fathers (fathers: 0–21.8%; mothers: 0–48.2%), implying that the intervention was more likely to produce changes in mothers’ parenting relative to fathers’ parenting. The *Moderate positive* parenting fathers in the control group at baseline were significantly more likely to move to the *Coercive* group than fathers in the treatment group (difference in transition = 21.8%, *p* = 0.017). For mothers, relative to the control group, those who were in the treatment group were more likely to move from the *Moderate positive* to the *High positive* group (difference in transition = 48.2%, *p* = 0.000) and, similar to fathers, those who were in the control group were more likely to move from *Moderate positive* to *Coercive* parenting group than those in the intervention group (difference in transition = 37.1%, *p* = 0.036). Also, there was a trend for mothers in the intervention group to be more likely to move from *Coercive* to *High positive* parenting relative to the control group (difference in transition = 21.2%, *p* = 0.058). In order to examine family-level change, the latent classes were extracted and classified parents who both transitioned in the same direction (e.g., to a higher positive group, to a lower positive parenting group). Out of 39 families who moved together to a higher positive parenting group, 29 families (74.4%) were involved in the intervention group, and two families in which parents moved together to a lower parenting group were all in the control group.

**Table 2:** Intervention-related changes in transition probabilities

**Fathers**		**1-year follow-up (T3)**		
	**Baseline (T1)**	*High positive*	*Moderate positive*	*Coercive*
Control	*High positive*	0.66	0.34	0.00
	*Moderate positive*	0.15	0.39	0.46
	*Coercive*	0.25	0.76	0.00
ADAPT	*High positive*	0.85	0.15	0.00
	*Moderate positive*	0.30	0.46	0.24
	*Coercive*	0.10	0.71	0.20
Difference	*High positive*	− 0.19	0.19	0.00
	*Moderate positive*	− 0.15	− 0.07	0.22^*^
	*Coercive*	0.15	0.05	− 0.20

**Mothers**		**1-year follow-up (T3)**		
	**Baseline (T1)**	*High positive*	*Moderate positive*	*Coercive*
Control	*High positive*	0.76	0.24	0.00
	*Moderate positive*	0.13	0.50	0.37
	*Coercive*	0.25	0.75	0.00
ADAPT	*High positive*	0.95	0.05	0.00
	*Moderate positive*	0.61	0.38	0.00
	*Coercive*	0.47	0.52	0.02
Difference	*High positive*	− 0.19	0.19	0.00
	*Moderate positive*	− 0.48^***^	0.11^**^	0.37^*^
	*Coercive*	− 0.21^+^	0.23	− 0.02

+ *p* < .10; *p < .05; **p < .01; ***p < .00

Finally, transition pathways were examined for differences in the distal outcome of child adjustment behaviors. All possible transition pathways were compared for each outcome. The Wald tests, conducted using the whole structural model including intervention status, indicated significant differences in child outcomes based on parenting transitions. For externalizing behaviors, children of fathers who remained in the *High positive* group for both time points had significantly lower child externalizing behavior compared to those who moved from the *High positive* to the *Coercive* parenting group (χ^2^(1) = 24.64, *p* = 0.000). For mothers, those who transitioned from the *Coercive* to the *High positive* parenting group had children with significantly lower externalizing problems than those who transitioned from the *Coercive* to the *Moderate positive* parenting group (χ^2^(1) = 11.358, *p* = 0.045). No other significant differences in child externalizing were observed for other transition groups. For internalizing behaviors, fathers who moved either to *High positive* or *Moderate positive* parenting from the *Coercive* parenting group showed significantly lower child internalizing problems compared to those who stayed in the *Coercive* parenting group (χ^2^_Coercive-High positive_ (1) = 15.942, *p* = 0.000; χ^2^_Coercive-Moderate positive_ (1) = 13.775, *p* = 0.000). However, transitions in mothers' parenting did not show significant differences in child internalizing behaviors.

## Discussion

The study examined longitudinal heterogeneity in mothers’ and fathers’ parenting practices and the effects of a preventive parenting intervention for military families. Our findings revealed significant heterogeneity of parenting behaviors among families who have experienced military deployments. Using five core parenting domains from an observed family interaction task, three meaningful subgroups of mothers and fathers were identified: *High positive*, *Moderate positive*, and *Coercive* parenting skills. *Coercive* parenting groups were characterized by high levels of harsh discipline in the interaction tasks. Past literature investigating the ADAPT intervention has shown a significant overall effect on parenting and parent mental health for mothers but not for fathers (Gewirtz et al., 2018b; DeGarmo & Gewirtz, 2018). The current study extended previous evaluations to identify heterogeneity in changes in parenting that depended on parents’ baseline parenting profiles.

The LTA revealed several important findings. Participants in the ADAPT program, both mothers and fathers, were more likely to show improvement in their parenting practices than those who received services-as-usual. Specifically, a preventive effect was found for both fathers and mothers who began with levels of positive parenting practices generally typical for the sample (i.e., *Moderate positive* group). Without ADAPT, a significant number of these families began to demonstrate more dysfunctional and less adaptive parenting after one year (i.e., *Moderate positive* to *Coercive* group). ADAPT effectively prevented this decline and reduced the number of families whose parenting became less adaptive over time.

Consistent with prior research by Gewirtz et al. (2016, 2018b), our results support that the treatment increased the likelihood of parents transitioning toward positive parenting, specifically for mothers. Notably, only mothers showed a significant difference in transition probabilities across intervention and control groups in movement from the *Moderate positive* to *High positive* parenting. Specifically, 61.4% of mothers in the ADAPT intervention group transitioned from *Moderate positive* to *High positive* parenting, a significant improvement compared to 13.2% in the control group. Moreover, there was a marginal effect on the movement from the *Coercive* to *High positive* parenting group for mothers as well. These changes indicate small to substantial shifts in parenting practices. On the other hand, mothers in the control group, who exhibited typical parenting at baseline, were more likely to remain in the *Moderate positive* parenting group. It is important to highlight that mothers who show typical levels of parenting behavior for this population are the ones most likely to benefit in terms of improved parenting. However, these findings also suggest that a considerable number of families may not experience such transitions, highlighting the need for ongoing support and tailored interventions.

The current results did not fully support the hypothesis that individuals with greater room for improvement would benefit more. Mothers with moderate levels of positive parenting tended to benefit more than those who had lower parenting skills marked by higher use of harsh discipline strategies. This is inconsistent with some existing literature examining risk as a moderator of response. For example, van Aar and colleagues (2019) found that parents exhibiting harsh and inconsistent parenting derived more intervention benefits from a parenting program relative to those parents who exhibited more adaptive parenting strategies. Piehler and colleagues (2022) showed that a higher risk profile characterized by maladaptive parenting and high externalizing child behaviors benefited most from a family-based intervention relative to lower risk profiles. Differences in our findings may be attributed to variations in the parenting programs used (e.g., Incredible Years, Early Risers), target populations (e.g., high risk of child disruptive behavior), and the combination of parenting and children’s problem behaviors as indicators. The differences observed in the current findings may also relate in part to linearity assumptions made within most previous single-variable moderator research. Our findings suggest a non-linear relationship between baseline risk and intervention response, with middle-risk individuals showing a greater response relative to low- and high-risk individuals that would not be captured by standard linear models. There is limited research in the literature that looks at parenting as a moderator for an intervention response.

Interestingly, the ADAPT parenting intervention supported a preventive effect for both parents in reducing the likelihood of developing coercive parenting. The parents in the control group were more likely to move toward the *Coercive* group involving higher harsh discipline than those in the intervention group. The percentage of mothers and fathers moving from *Moderate positive* to *Coercive parenting* was significantly lower in the ADAPT program, with only 0.2% of mothers making this transition compared to 37.3% in the control group, and 24.4% of fathers in the ADAPT program compared to 46.2% in the control group. These results indicate a moderate effect of ADAPT in preventing the deterioration of parenting practices.

Father intervention effects provide unique evidence that the parenting program is effective in its preventive role for fathers who had not shown a reliable intervention effect in previous analysis of ADAPT studies (Gewirtz et al., 2019). This protective effect may explain why intervention effects on fathers have been harder to detect. Preventive effects that occur only within a specific subgroup are likely to be obscured with a variable-centered approach. This highlights the importance of identifying heterogeneity within parents and the value of a person-centered approach to understanding changes in parenting. Our findings illustrate that a preventive intervention may help offset what may be a typical pattern of parenting change for a subset of post-deployed military families. Without a parenting program, family processes may degrade for some families due to ongoing stressors such as mental health concerns.

Supporting the importance of parenting transitions, negative transitions were associated with higher child externalizing and internalizing behavior. This aligns with several meta-analyses demonstrating the link between parenting strategies and child adjustment (Pinquart, 2017; Rueger et al., 2016; Weymouth et al., 2016). For child externalizing behavior, children of fathers who moved from *High positive* to *Coercive* parenting skills had a higher average of externalizing problems at a two-year follow-up relative to those who did not make this transition. This association is consistent with results of a meta-analytic review indicating that poor paternal parenting was more strongly associated with child delinquency than poor maternal parenting (Hoeve et al., 2012). In the current study, mothers who showed the greatest improvement in their parenting practices had children with lower externalizing behaviors relative to those mothers who tended not to make improvements.

For child internalizing behavior, only fathers’ positive movement in parenting tended to be associated with reductions in child internalizing relative to those who maintained lower levels of positive parenting. Mothers’ transitions in parenting did not show any relationship with child internalizing behavior. This may indicate that a post-deployment paternal reduction in psychological control (i.e., manipulating child’s thoughts and feelings, conditional loving) and an increase in positive encouragement may be most effective in improving child internalizing behavior, as internalizing has shown a stronger relationship with parents’ psychological control than behavioral control (Gorostiaga et al., 2019). However, in this study, we did not measure psychological control; future studies should include measurement of this construct. Yet, some empirical studies have shown similar maternal parenting effects on internalizing symptoms (Rueger et al., 2016; Smokowski et al., 2015; Vazsonyi et al., 2021). To understand the variability of parental effects on a child’s internalizing symptoms, future studies should look at the moderating effect of parent gender between their parenting and child internalizing behavior.

Consequently, these results underscore the potential long-term benefits of such transitions through parenting programs, as changes in parents' profiles were meaningfully associated with distal child behavioral problems. While the improvements in mothers’ parenting were more notable, it is important to recognize the preventive impact on fathers' parenting. Both parents play essential roles in child development, and enhancements in paternal parenting can yield substantial benefits for children as positive transitions in fathers' parenting were related to reductions in child internalizing symptoms. Moreover, quality father involvement and coordinated parenting efforts have shown better child outcomes (Allport et al., 2018; Lee & Schoppe-Sullivan, 2017; McWayne et al., 2013; Teubert & Pinquart, 2010). Therefore, tailored activities or trauma-informed care before the intervention may be needed to support and engage fathers, particularly those with combat experience, to improve their parenting practices. A balanced approach that supports both mothers and fathers is necessary for fostering healthy family dynamics.

## Limitations

Several limitations of the current study should be noted. First, there is the possibility of bias in results due to the small sample size. Because LPA is an exploratory analysis to find unobserved groups within a sample, it is recommended to have a large sample size in order to have enough power to detect distinct subgroups; LTA also requires intensive computation as a mixture model and performs best with large sample sizes (Lanza et al., 2013). Although the sample of 336 military families is relatively large for this unique population, the three-class model may not fully capture the population's heterogeneity. Therefore, additional studies are needed to replicate the classes and examine the relationship between changes in parenting and child distal outcomes. Despite these limitations, our findings suggest the presence of three relatively consistent parenting classes and a potential relationship between changes in parenting and child maladjustment. Moreover, while intervention status was included in the model to compare long-term outcomes, it is important to note that the results indicate associations between the intervention and child behavior outcomes through changes in parenting profiles, rather than definitive direct causality.

Second, to facilitate the interpretation of class transitions, the LTA used a full invariance model across timepoints regardless of the variance in item-response probabilities. This LTA did not compare how the profiles were different across time but rather how the parents moved from one class to another. Again, future studies with a larger sample size can explore the extent of the variance across the two latent profiles at each time point. Yet, this study gives an important foundation to understand the actual movement of parents in the context of parenting.

Third, there is a potential for rater bias as the study relied only on an observational measure of parenting. Even though there is a lower risk of self-report bias in observational coding relative to self-report rating scales, the use of global coding still cannot eliminate the coder’s bias in their perception of the family interaction. For example, the coders could have given an overall high score for a particular family if they perceived a positive interaction in general. This could have caused the division of parenting profiles into three levels with overall relatively consistent skills rather than showing mixed parenting profiles. However, observations are more reliable estimates of change and robust measurements of parenting that can prevent expectancy bias (Patterson, 1982; Snyder et al., 2006). In future studies, parenting practices collected through multiple measures (i.e., self-reported parenting, physiological regulation) can be examined together with observational data to identify subgroups within the families.

Finally, a few limitations are important to note about the sample. As this study focused on those who were recently deployed, these findings cannot be generalized to other parents who participate in parent training programs. Also, a lack of diversity in the sample should be noted; the majority of participants were white middle-income families from a Midwestern state. Therefore, future studies examining the replicability of the findings with more diverse families will be important.

## Conclusions

The current study demonstrates that the ADAPT program effectively supports improvements in parenting practices among military families, particularly for mothers who exhibit typical levels of positive parenting. However, it is crucial to recognize the preventive effects observed in fathers' parenting, suggesting that the ADAPT program may be more effective for military parents who already have some positive parenting practices. This implies that the program might benefit from additional components to better support post-deployed military families with highly dysfunctional parent-child interactions – for example, treatment referrals, or additional individual support in ADAPT skill building from an ADAPT clinician. Clinicians working with deployed families should consider parents’ baseline parenting skills. If harsh parenting skills are noticed, additional pre-program services could be utilized to help these parents benefit most from the program.

## Supplementary Information

Below is the link to the electronic supplementary material.

Supplementary file1 (DOCX 36 KB)

## Data Availability

The data that support the findings of this study are available from the corresponding author upon request.

## Footnotes

1 ADAPT has subsequently been adapted and tested via multiple delivery formats including online and telehealth.

## References

[CR1] Akaike, A., Ohno, Y., Sasa, M., & Takaori, S. (1987). Excitatory and inhibitory effects of dopamine on neuronal activity of the caudate nucleus neurons in vitro. *Brain Research,**418*(2), 262–272. 10.1016/0006-8993(87)90094-1 2890403 10.1016/0006-8993(87)90094-1

[CR2] Allport, B. S., Johnson, S., Aqil, A., Labrique, A. B., Nelson, T., Angela, K. C., ..., & Marcell, A. V. (2018). Promoting father involvement for child and family health. *Academic Pediatrics,**18*(7), 746–753. 29653255 10.1016/j.acap.2018.03.011

[CR3] Asnaani, A., Reddy, M. K., & Shea, M. T. (2014). The impact of PTSD symptoms on physical and mental health functioning in returning veterans. *Journal of Anxiety Disorders,**28*(3), 310–317. 24647406 10.1016/j.janxdis.2014.01.005

[CR4] Barlow, J., & Stewart-Brown, S. (2000). Behavior problems and group-based parent education programs. *Journal of Developmental and Behavioral Pediatrics,**21*(4), 356–370. 11064964 10.1097/00004703-200010000-00007

[CR5] Belsky, J. (1984). The determinants of parenting: A process model. *Child Development,**55*(1), 83–96. 10.2307/1129836 6705636 10.1111/j.1467-8624.1984.tb00275.x

[CR6] Byrnes, H. F., & Miller, B. A. (2012). The relationship between neighborhood characteristics and effective parenting behaviors: The role of social support. *Journal of Family Issues,**33*(12), 1658–1687. 10.1177/0192513X12437693 23794774 10.1177/0192513X12437693 PMC3685862

[CR7] Chesmore, A. A., Piehler, T. F., & Gewirtz, A. H. (2018). PTSD as a moderator of a parenting intervention for military families. *Journal of Family Psychology,**32*(1), 123–134. 29283597 10.1037/fam0000366 PMC5854523

[CR8] Collins, L. M., & Lanza, S. T. (2009). *Latent class and latent transition analysis: With applications in the social, behavioral, and health sciences* (Vol. 718). USA: John Wiley & Sons.

[CR9] Collins, L. M., & Wugalter, S. E. (1992). Latent class models for stage-sequential dynamic latent variables. *Multivariate Behavioral Research,**27*(1), 131–157.

[CR10] Connell, A., Bullock, B. M., Dishion, T. J., Shaw, D., Wilson, M., & Gardner, F. (2008). Family intervention effects on co-occurring early childhood behavioral and emotional problems: A latent transition analysis approach. *Journal of Abnormal Child Psychology,**36*(8), 1211–1225. 10.1007/s10802-008-9244-6 18473160 10.1007/s10802-008-9244-6 PMC2710140

[CR11] Creech, S. K., Hadley, W., & Borsari, B. (2014). The impact of military deployment and reintegration on children and parenting: A systematic review. *Professional Psychology: Research and Practice,**45*(6), 452–464. 25844014 10.1037/a0035055 PMC4383395

[CR12] de Graaf, I., Speetjens, P., Smit, F., De Wolff, M., & Tavecchio, L. (2008). Effectiveness of the Triple P Positive Parenting Program on parenting: A meta-analysis. *Family Relations,**57*(5), 553–566. 10.1111/j.1741-3729.2008.00522.x 10.1177/0145445508317134 18475003

[CR13] DeGarmo, D. S., & Gewirtz, A. H. (2018). A recovery capital and stress-buffering model for post-deployed military parents. *Frontiers in Psychology,**9*, 14. 30337896 10.3389/fpsyg.2018.01832 PMC6180167

[CR14] Epstein, R. A., Fonnesbeck, C., Potter, S., Rizzone, K. H., & McPheeters, M. (2015). Psychosocial interventions for child disruptive behaviors: A meta-analysis. *Pediatrics,**136*(5), 947–960. 10.1542/peds.2015-2577 26482672 10.1542/peds.2015-2577

[CR15] Fairchild, A. J., & MacKinnon, D. P. (2014). Using mediation and moderation analyses to enhance prevention research. In H. Petras (Ed.), *Sloboda Z. *Defining Prevention Science. Advances in Prevention Science. Boston, MA: Springer.

[CR16] Fletcher, R., Freeman, E., & Matthey, S. (2011). The impact of behavioural parent training on fathers’ parenting: A meta-analysis of the Triple P-Positive Parenting Program. *Fathering,**9*, 291–312. 10.3149/fth.0903.291

[CR17] Forgatch, M. S., Knutson, N., & Mayne, T. (1992). *Coder impressions of ODS lab tasks. *Eugene, OR: Oregon Social Learning Center.

[CR18] Forgatch, M. S., & DeGarmo, D. S. (1999). Parenting through change: An effective prevention program for single mothers. *Journal of Consulting and Clinical Psychology,**67*, 711–724. 10535238 10.1037//0022-006x.67.5.711

[CR19] Forgatch, M. S., & Patterson, G. R. (2010). Parent Management Training—Oregon Model: An intervention for antisocial behavior in children and adolescents. In J. R. Weisz & A. E. Kazdin (Eds.), *Evidence-based Psychotherapies for Children and Adolescents* (pp. 159–177). The Guilford Press.

[CR20] Gewirtz, A. H., Degarmo, D. S., & Zamir, O. (2018a). Testing a military family stress model. *Family Process, 57*(2), 415–431. 10.1111/famp.12282 10.1111/famp.12282 PMC6788861 28299783

[CR21] Gewirtz, A. H., Degarmo, D. S., & Zamir, O. (2018b). After Deployment, Adaptive Parenting Tools One-year outcomes of an evidence-based parenting program for military families following deployment. *Prevention Science*. 10.1007/s11121-017-0839-4 28913717 10.1007/s11121-017-0839-4 PMC5854502

[CR22] Gewirtz, A. H., DeGarmo, D. S., & Zamir, O. (2016). Effects of a military parenting program on parental distress and suicidal ideation: After deployment adaptive parenting tools. *Suicide and Life-Threatening Behavior,**46*, S23–S31. 10.1111/sltb.12255 27094107 10.1111/sltb.12255 PMC5113712

[CR23] Gewirtz, A. H., Pinna, K. L., Hanson, S. K., & Brockberg, D. (2014). Promoting parenting to support reintegrating military families: After deployment, adaptive parenting tools. *Psychological Services,**11*(1), 31. 10.1037/a0034134 24564441 10.1037/a0034134 PMC4030517

[CR24] Gewirtz, A. H., Polusny, M. A., DeGarmo, D. S., Khaylis, A., & Erbes, C. R. (2010). Posttraumatic stress symptoms among National Guard soldiers deployed to Iraq: Associations with parenting behaviors and couple adjustment. *Journal of Consulting and Clinical Psychology,**78*(5), 599. 20873896 10.1037/a0020571 PMC3073229

[CR25] Gewirtz, A. H., Snyder, J., Zamir, O., Zhang, J., & Zhang, N. (2019). Effects of the After Deployment: Adaptive Parenting Tools (ADAPT) intervention on fathers and their children: A moderated mediation model. *Development and Psychopathology,**31*(5), 1837–1849. 31718738 10.1017/S0954579419001238

[CR26] Giff, S. T., Renshaw, K. D., & Allen, E. S. (2019). Post-deployment parenting in military couples: Associations with service members’ PTSD symptoms. *Journal of Family Psychology,**33*(2), 166. 30451514 10.1037/fam0000477

[CR27] Gorman, G. H., Eide, M., & Hisle-Gorman, E. (2010). Wartime military deployment and increased pediatric mental and behavioral health complaints. *Pediatrics,**126*(6), 1058–1066. 21059715 10.1542/peds.2009-2856

[CR28] Gorostiaga, A., Aliri, J., Balluerka, N., & Lameirinhas, J. (2019). Parenting styles and internalizing symptoms in adolescence: A systematic literature review. *International Journal of Environmental Research and Public Health,**16*(17), 3192. 31480548 10.3390/ijerph16173192 PMC6747480

[CR29] Gross, D., Fogg, L., Webster-Stratton, C., Garvey, C. W. J., & Grady, J. (2003). Parent training with multiethnic families of toddlers in day care in low-income urban communities. *Journal of Consulting and Clinical Psychology,**71*(2), 261–278. 12699021 10.1037/0022-006x.71.2.261

[CR30] Hoeve, M., Stams, G. J. J., Van der Put, C. E., Dubas, J. S., Van der Laan, P. H., & Gerris, J. R. (2012). A meta-analysis of attachment to parents and delinquency. *Journal of Abnormal Child Psychology,**40*(5), 771–785. 10.1007/s10802-011-9608-1 22278802 10.1007/s10802-011-9608-1 PMC3375078

[CR31] Hutchings, J., Bywater, T., Daley, D., Gardner, F., Whitaker, C., Jones, K., ..., & Edwards, R. T. (2007). Parenting intervention in Sure Start services for children at risk of developing conduct disorder: pragmatic randomized controlled trial. *BMJ,**334*(7595), 678. 17350966 10.1136/bmj.39126.620799.55 PMC1839187

[CR32] Jacobson, I. G., Ryan, M. A. K., Hooper, T. I., Smith, T. C., Amoroso, P. J., Boyko, E. J., et al. (2008). Alcohol use and alcohol-related problems before and after military combat deployment. *Journal of the American Medical Association,**300*, 663–675. 18698065 10.1001/jama.300.6.663 PMC2680184

[CR33] Julian, M. M., Muzik, M., Kees, M., Valenstein, M., & Rosenblum, K. L. (2018). Strong Military Families intervention enhances parenting reflectivity and representations in families with young children. *Infant Mental Health Journal,**39*(1), 106–118. 29286541 10.1002/imhj.21690

[CR34] Khaylis, A., Polusny, M. A., Erbes, C. R., Gewirtz, A., & Rath, M. (2011). Posttraumatic stress, family adjustment, and treatment preferences among National Guard soldiers deployed to OEF/OIF. *Military Medicine,**176*(2), 126–131. 21366071 10.7205/milmed-d-10-00094

[CR35] Kim, E., Cain, K. C., & Webster-Stratton, C. (2008). The preliminary effect of a parenting program for Korean American mothers: A randomized controlled experimental study. *International Journal of Nursing Studies,**45*(9), 1261–1273. 17996239 10.1016/j.ijnurstu.2007.10.002 PMC2564289

[CR36] Knutson, N. M., Forgatch, M. S., Rains, L. A., Sigmarsdóttir, M., & Domenech Rodríguez, M. (2019). *Fidelity of implementation rating system (FIMP): The manual for GenerationPMTO* (3rd ed.). Implementation Sciences International.

[CR37] Lanza, S. T., Bray, B. C., & Collins, L. M. (2013). An introduction to latent class and latent transition analysis. In J. A. Schinka, W. F. Velicer, & I. B. Weiner (Eds.), *Handbook of Psychology: Research Methods in Psychology* (pp. 691–716). John Wiley & Sons Inc.

[CR38] Lee, J. K., & Schoppe-Sullivan, S. J. (2017). Resident fathers’ positive engagement, family poverty, and change in child behavior problems. *Family Relations,**66*(3), 484–496.

[CR39] Leijten, P., Raaijmakers, M. A., de Castro, B. O., & Matthys, W. (2013). Does socioeconomic status matter? A meta-analysis on parent training effectiveness for disruptive child behavior. *Journal of Clinical Child & Adolescent Psychology,**42*(3), 384–392. 23461526 10.1080/15374416.2013.769169

[CR40] Lester, P., Aralis, H., Sinclair, M., Kiff, C., Lee, K. H., Mustillo, S., & Wadsworth, S. M. (2016). The impact of deployment on parental, family and child adjustment in military families. *Child Psychiatry & Human Development,**47*(6), 938–949. 26797704 10.1007/s10578-016-0624-9

[CR41] Lo, Y., Mendell, N. R., & Rubin, D. B. (2001). Testing the number of components in a normal mixture. *Biometrika,**88*(3), 767–778. 10.1093/biomet/88.3.767

[CR42] Mackesy-Amiti, M. E., Finnegan, L., Ouellet, L. J., Golub, E. T., Hagan, H., Hudson, S. M., ..., & Garfein, R. S. (2013). Peer-education intervention to reduce injection risk behaviors benefits high-risk young injection drug users: A latent transition analysis of the CIDUS 3/DUIT study. *AIDS and Behavior,**17*, 2075–2083. 23142857 10.1007/s10461-012-0373-0 PMC3672505

[CR43] Mansfield, A. J., Kaufman, J. S., Marshall, S. W., Gaynes, B. N., Morrissey, J. P., & Engel, C. C. (2010). Deployment and the use of mental health services among US Army wives. *New England Journal of Medicine,**362*(2), 101–109. 20071699 10.1056/NEJMoa0900177

[CR44] McGrath, J. E., & Tschan, F. (2004). *Temporal matters in social psychology: Examining the role of time in the lives of groups and individuals*. American Psychological Assocation. 10.1037/10659-000

[CR45] McWayne, C., Downer, J. T., Campos, R., & Harris, R. D. (2013). Father involvement during early childhood and its association with children’s early learning: A meta-analysis. *Early Education & Development,**24*(6), 898–922.

[CR46] Menting, A. T., de Castro, B. O., & Matthys, W. (2013). Effectiveness of the Incredible Years parent training to modify disruptive and prosocial child behavior: A meta-analytic review. *Clinical Psychology Review,**33*(8), 901–913. 10.1016/j.cpr.2013.07.006 23994367 10.1016/j.cpr.2013.07.006

[CR47] Mmari, K., Roche, K. M., Sudhinaraset, M., & Blum, R. (2009). When a parent goes off to war: Exploring the issues faced by adolescents and their families. *Youth and Society,**40*(4), 455–475. 10.1177/0044118X08327873

[CR48] Niec, L. N., Barnett, M. L., Gering, C. L., Triemstra, K., & Solomon, D. T. (2015). Differences in mothers’ and fathers’ readiness for change in parent training. *Child & Family Behavior Therapy,**37*(3), 224–235. 10.1080/07317107.2015.1071980

[CR49] Nylund, K., Bellmore, A., Nishina, A., & Graham, S. (2007). Subtypes, severity, and structural stability of peer victimization: What does latent class analysis say?. *Child development, 78*(6), 1706–1722. 10.1111/j.1467-8624.2007.01097.x 10.1111/j.1467-8624.2007.01097.x 17988316

[CR50] Paley, B., Lester, P., & Mogil, C. (2013). Family systems and ecological perspectives on the impact of deployment on military families. *Clinical Child and Family Psychology Review,**16*(3), 245–265. 23760926 10.1007/s10567-013-0138-y

[CR51] Panter-Brick, B., & A., Eggerman, M., McAllister, F., Pruett, K., & Leckman, J. F. (2014). Practitioner Review: Engaging fathers – Recommendations for a game change in parenting interventions based on a systematic review of the global evidence. *Journal of Child Psychology and Psychiatry and Allied Disciplines.,**55*(11), 1187–1212. 24980187 10.1111/jcpp.12280 PMC4277854

[CR52] Patterson, G. R. (1982).* Coercive family process*. Eugene, OR: Castalia.

[CR53] Patterson, G. R. (2005). The next generation of PMTO models. *The Behavior Therapist,**28*(2), 27–33.

[CR54] Pelham, W. E., Dishion, T. J., Tein, J. Y., Shaw, D. S., & Wilson, M. N. (2017). What doesn’t work for whom? Exploring heterogeneity in responsiveness to the family check-up in early childhood using a mixture model approach. *Prevention Science,**18*(8), 911–922. 28550456 10.1007/s11121-017-0805-1 PMC5693624

[CR55] Piehler, T. F., Zhang, J., Bloomquist, M. L., & August, G. J. (2022). Parent and child risk profiles as predictors of response to a conduct problem preventive intervention. *Prevention Science,**23*(7), 1308–1320. 35486296 10.1007/s11121-022-01374-4

[CR56] Pinquart, M. (2017). Associations of parenting dimensions and styles with externalizing problems of children and adolescents: An updated meta-analysis. *Developmental Psychology,**53*(5), 873. 10.1037/dev0000295 28459276 10.1037/dev0000295

[CR57] Polusny, M. A., Erbes, C. R., Murdoch, M., Arbisi, P. A., Thuras, P., & Rath, M. B. (2011). Prospective risk factors for new-onset post-traumatic stress disorder in National Guard soldiers deployed to Iraq. *Psychological Medicine,**41*(4), 687–698. 21144108 10.1017/S0033291710002047

[CR58] Reyno, S. M., & McGrath, P. J. (2006). Predictors of parent training efficacy for child externalizing behavior problems – A meta-analytic review. *Journal of Child Psychology and Psychiatry, and Allied Disciplines,**47*(1), 99–111. 16405646 10.1111/j.1469-7610.2005.01544.x

[CR59] Reynolds, C. R., & Kamphaus, R. W. (2004). *Behavior assessment system for children (BASC-2)*. American Guidance Service.

[CR60] Roberts, T. J., & Ward, S. E. (2011). Using latent transition analysis in nursing research to explore change over time. *Nursing Research,**60*(1), 73. 21127448 10.1097/NNR.0b013e3182001c63 PMC3108848

[CR61] Rueger, S. Y., Malecki, C. K., Pyun, Y., Aycock, C., & Coyle, S. (2016). A meta-analytic review of the association between perceived social support and depression in childhood and adolescence. *Psychological Bulletin,**142*(10), 1017. 10.1037/bul0000058 27504934 10.1037/bul0000058

[CR62] Sanders, M. R., Markie-Dadds, C., Tully, L. A., & Bor, W. (2000). The Triple P-positive parenting program: A comparison of enhanced, standard, and self-directed behavioral family intervention for parents of children with early onset conduct problems. *Journal of Consulting and Clinical Psychology,**68*(4), 624. 10965638

[CR63] Schwarz, G. (1978). Estimating the dimension of a model. *The Annals of Statistics, 6*(2), 461–464. http://www.jstor.org/stable/2958889

[CR64] Sclove, S. L. (1987). Application of model-selection criteria to some problems in multivariate analysis. *Psychometrika,**52*, 333–343. 10.1007/BF02294360

[CR65] Scott, S., Sylva, K., Doolan, M., Price, J., Jacobs, B., Crook, C., & Landau, S. (2010). Randomised controlled trial of parent groups for child antisocial behaviour targeting multiple risk factors: The SPOKES project. *Journal of Child Psychology and Psychiatry,**51*(1), 48–57. 10.1111/j.1469-7610.2009.02127.x 19732250 10.1111/j.1469-7610.2009.02127.x

[CR66] Smokowski, P. R., Bacallao, M. L., Cotter, K. L., & Evans, C. B. (2015). The effects of positive and negative parenting practices on adolescent mental health outcomes in a multicultural sample of rural youth. *Child Psychiatry & Human Development,**46*(3), 333–345. 24880498 10.1007/s10578-014-0474-2

[CR67] Snyder, J., Reid, J., Stoolmiller, M., Howe, G., Brown, H., Dagne, G., & Cross, W. (2006). The role of behavior observation in measurement systems for randomized prevention trials. *Prevention Science,**7*(1), 43–56. 10.1007/s11121-005-0020-3 10.1007/s11121-005-0020-3 16572301

[CR68] Teubert, D., & Pinquart, M. (2010). The association between coparenting and child adjustment: A meta-analysis. *Parenting,**10*(4), 286–307. 10.1080/15295192.2010.492040

[CR69] The Clearinghouse Continuum of Evidence (2023, February 1). *Adaptive Parenting Tools (ADAPT)*. Retrieved September 6, 2023, from https://www.continuum.militaryfamilies.psu.edu/program/fact_sheet_974

[CR70] Thomas, R., & Zimmer-Gembeck, M. J. (2007). Behavioral outcomes of parent-child interaction therapy and Triple P—Positive Parenting Program: A review and meta-analysis. *Journal of Abnormal Child Psychology,**35*(3), 475–495. 17333363 10.1007/s10802-007-9104-9

[CR71] Tiano, J. D., & McNeil, C. B. (2005). The inclusion of fathers in behavioral parent training: A critical evaluation. *Child & Family Behavior Therapy,**27*(4), 1–28.

[CR72] Turney, K. (2015). Beyond average effects: Incorporating heterogeneous treatment effects into family research. *Journal of Family Theory & Review,**7*(4), 468–481.

[CR73] van Aar, J., Leijten, P., de Castro, B. O., Weeland, J., Matthys, W., Chhangur, R., & Overbeek, G. (2019). Families who benefit and families who do not: Integrating person-and variable-centered analyses of parenting intervention responses. *Journal of the American Academy of Child & Adolescent Psychiatry,**58*(10), 993–1003. 10.1016/j.jaac.2019.02.004 30768388 10.1016/j.jaac.2019.02.004

[CR74] Vazsonyi, A. T., Ksinan, A. J., Javakhishvili, M., Scarpate, J. M., & Kahumoku-Fessler, E. (2021). Links between parenting and internalizing and externalizing problems: Cross-cultural evidence from ten countries. *Child Psychiatry & Human Development,**53*(4), 667–683. 10.1007/s10578-021-01153-2 33751285 10.1007/s10578-021-01153-2

[CR75] Weeland, J., Helmerhorst, K. O., & Lucassen, N. (2021). Understanding differential effectiveness of behavioral parent training from a family systems perspective: Families are greater than “some of their parts.” *Journal of Family Theory & Review,**13*(1), 34–57.

[CR76] Weeland, J., Leijten, P., Orobio de Castro, B., Menting, A., Overbeek, G., Raaijmakers, M., & Matthys, W. (2023). Exploring parenting profiles to understand who benefits from the incredible years parenting program. *Prevention Science, 24*(2), 259–270. 10.1007/s11121-022-01364-6 10.1007/s11121-022-01364-6 PMC9938070 35305230

[CR77] Weymouth, B. B., Buehler, C., Zhou, N., & Henson, R. A. (2016). A meta-analysis of parent–adolescent conflict: Disagreement, hostility, and youth maladjustment. *Journal of Family Theory & Review,**8*(1), 95–112. 10.1111/jftr.12126

[CR78] Wymbs, B. T. (2011). Mechanisms underlying the influence of disruptive child behavior on interparental communication. *Journal of Family Psychology,**25*(6), 873–884. 21875193 10.1037/a0025372 PMC3547400

[CR79] Zhang, J., Zhang, N., Piehler, T. F., & Gewirtz, A. H. (2023). Emotion regulation difficulties in military fathers magnify their benefit from a parenting program. *Prevention Science,**24*(2), 237–248. 34333734 10.1007/s11121-021-01287-8

[CR80] Zhang, N., Lee, S. K., Zhang, J., Piehler, T., & Gewirtz, A. (2020). Growth trajectories of parental emotion socialization and child adjustment following a military parenting intervention: A randomized controlled trial. *Developmental Psychology, 56*(3), 652. 10.1037/dev0000837 10.1037/dev0000837 PMC7041849 32077731

[CR81] Zhang, N., Zhang, J., Gewirtz, A. H., & Piehler, T. F. (2018). Improving parental emotion socialization in military families: Results of a randomized controlled trial. *Journal of Family Psychology,**32*(8), 1046. 10.1037/fam0000461 30102051 10.1037/fam0000461 PMC6392434

