# Supplementary material for: Parenting Profiles in Military Families: Intervention-Related Transitions and Relationships to Child Adjustment
Source: Prev Sci. 2024 Sep 16;25(7):1040–52. doi: 10.1007/s11121-024-01721-7 (PMC11519299; doi:10.1007/s11121-024-01721-7)
Supplement: Supplementary file 1 — Supplementary file1 (DOCX 36 KB) [file 11121_2024_1721_MOESM1_ESM.docx]

**Supplemental Tables**

| Supplemental Table 1. Descriptive statistics of Fathers’ and Mothers’ Study Variables | | | | | | | | | | | | | | | | | | | | | | | | | | | | | | | |
| --- | --- | --- | --- | --- | --- | --- | --- | --- | --- | --- | --- | --- | --- | --- | --- | --- | --- | --- | --- | --- | --- | --- | --- | --- | --- | --- | --- | --- | --- | --- | --- |
| Study variables | | 1 | 2 | 3 | | 4 | | 5 | | 6 | | 7 | | 8 | | 9 | | 10 | | 11 | | 12 | | 13 | | 14 | | 15 | | 16 | |
| 1. Treat | | 1 | .03 | | .02 | | -.03 | | -.10 | | .09 | | -.04 | | .15^*^ | | .20^**^ | | .06 | | .01 | | .00 | | -.01 | | -.11 | | -.04 | | -.05 |
| 1. Mar. Status | | -.10 | 1 | | .18^**^ | | .20^**^ | | .02 | | .09 | | -.24^**^ | | .14^*^ | | .09 | | -.04 | | .00 | | -.22^**^ | | -.11 | | -.13^*^ | | -.04 | | -.05 |
| 1. PSO T1 | | -.01 | .11 | | 1 | | .53^**^ | | .20^**^ | | .09 | | -.32^**^ | | .33^**^ | | .27^**^ | | .01 | | -.01 | | -.26^**^ | | -.25^**^ | | -.13 | | -.01 | | .01 |
| 1. PINV T1 | | -.04 | .00 | | .47^**^ | | 1 | | .46^**^ | | .24^**^ | | -.44^**^ | | .20^**^ | | .34^**^ | | .14^*^ | | .04 | | -.29^**^ | | -.12^*^ | | -.05 | | .03 | | .06 |
| 1. ENC T1 | | -.05 | -.09 | | .23^**^ | | .62^**^ | | 1 | | .31^**^ | | -.20^**^ | | .12 | | .32^**^ | | .36^**^ | | .20^**^ | | -.11 | | .13^*^ | | .12 | | -.01 | | .09 |
| 1. MON T1 | | .02 | -.08 | | .10 | | .39^**^ | | .37^**^ | | 1 | | -.26^**^ | | .04 | | .05 | | .09 | | .11 | | .08 | | .01 | | .01 | | -.07 | | .00 |
| 1. DIS T1 | | -.01 | .12 | | -.26^**^ | | -.29^**^ | | -.15^*^ | | -.11 | | 1 | | -.16^*^ | | -.22^**^ | | .00 | | .00 | | .33^**^ | | .15^*^ | | .01 | | -.05 | | .01 |
| 1. PSO T3 | | .12 | .06 | | .26^**^ | | .25^**^ | | .16^*^ | | .16^*^ | | -.06 | | 1 | | .54^**^ | | .13 | | .11 | | -.31^**^ | | -.06 | | -.12 | | .04 | | -.01 |
| 1. PINV T3 | | .09 | .00 | | .24^**^ | | .42^**^ | | .34^**^ | | .29^**^ | | -.08 | | .54^**^ | | 1 | | .50^**^ | | .21^**^ | | -.30^**^ | | .00 | | -.04 | | .05 | | .02 |
| 1. ENC T3 | | -.05 | -.04 | | .03 | | .31^**^ | | .47^**^ | | .26^**^ | | .01 | | .28^**^ | | .52^**^ | | 1 | | .21^**^ | | .07 | | .15^*^ | | .11 | | -.08 | | .02 |
| 1. MON T3 | | .07 | .04 | | .05 | | .21^**^ | | .18^*^ | | .29^**^ | | -.11 | | .11 | | .30^**^ | | .25^**^ | | 1 | | .00 | | .08 | | .04 | | -.05 | | -.06 |
| 1. DIS T3 | | .01 | -.07 | | -.16^*^ | | -.25^**^ | | -.18^*^ | | -.07 | | .29^**^ | | -.26^**^ | | -.51^**^ | | -.23^**^ | | -.30^**^ | | 1 | | .10 | | .23^**^ | | -.01 | | .08 |
| 1. Ext T1 | | -.04 | -.14^*^ | | -.17^**^ | | .01 | | .09 | | .04 | | .15^*^ | | -.06 | | .08 | | .16^*^ | | .04 | | .00 | | 1 | | .48^**^ | | .11 | | .16^**^ |
| 1. Ext T4 | | -.10 | -.06 | | -.16^*^ | | -.17^*^ | | .00 | | -.11 | | .14 | | -.12 | | -.14 | | .00 | | -.09 | | .21^**^ | | .51^**^ | | 1 | | .25^**^ | | .47^**^ |
| 1. Int T1 | | -.08 | .00 | | -.06 | | .03 | | .00 | | .03 | | -.09 | | -.02 | | .04 | | -.07 | | -.03 | | .02 | | .12 | | .20^**^ | | 1 | | .65^**^ |
| 1. Int T4 | | -.09 | -.05 | | -.03 | | .06 | | .04 | | -.01 | | .03 | | -.06 | | -.04 | | -.07 | | -.04 | | .06 | | .22^**^ | | .45^**^ | | .62^**^ | | 1 |
| Father | Mean | 0.62 | 0.89 | | 2.50 | | 3.39 | | 2.70 | | 3.15 | | 1.30 | | 2.81 | | 3.54 | | 2.64 | | 3.20 | | 1.23 | | 57.90 | | 50.88 | | 51.62 | | 51.16 |
|  | SD | 0.49 | 0.31 | | 0.62 | | 0.53 | | 0.77 | | 0.94 | | 0.35 | | 0.66 | | 0.53 | | 0.64 | | 0.84 | | 0.38 | | 14.31 | | 8.37 | | 9.63 | | 9.27 |
| Mother | Mean | 0.60 | 0.89 | | 2.56 | | 3.49 | | 2.66 | | 3.69 | | 1.37 | | 2.97 | | 3.67 | | 2.62 | | 3.62 | | 1.25 | | 57.96 | | 51.15 | | 51.71 | | 51.19 |
|  | SD | 0.49 | 0.32 | | 0.67 | | 0.46 | | 0.70 | | 0.78 | | 0.43 | | 0.69 | | 0.48 | | 0.67 | | 0.74 | | 0.37 | | 14.15 | | 8.53 | | 9.84 | | 9.79 |
| *Note.* PSO = Problem Solving; PINV = Positive Involvement; ENC = Encouragement; MON = Monitoring; DIS = inept discipline. Positive correlation shows fathers’ and inverse correlation shows mothers’ inter-correlations between study variables. Marital status was coded 1 (*married*) and 0 (*single*) and treatment status was coded 1 (*treatment*).  * p<.05, ** p<.01, ***p<.001 | | | | | | | | | | | | | | | | | | | | | | | | | | | | | | | |

| Supplemental Table 2. Absolute and Relative Fit Indices for Measurement Models at T1 and T3 | | | | | | | | | |
| --- | --- | --- | --- | --- | --- | --- | --- | --- | --- |
| *Group* | *Time* | *Classes* | *LL* | *AIC* | *BIC* | *aBIC* | *Entropy* | *LMR-LRT (p)* | |
| Fathers | T1 | 2 | -1157.07 | 2346.138 | 2404.006 | 2353.273 | 0.69 | 177.596 | 0.005 |
|  |  | 3 | -1115.39 | 2274.785 | 2354.354 | 2284.596 | 0.758 | 80.951 | 0.047 |
|  |  | 4 | -1093.77 | 2243.533 | 2344.802 | 2256.02 | 0.819 | 42.006 | 0.375 |
|  |  | 5 | -1074.48 | 2216.957 | 2339.928 | 2232.12 | 0.853 | 37.463 | 0.038 |
|  | T3 | 2 | -829.666 | 1691.331 | 1744.732 | 1694.036 | 0.977 | 183.977 | 0.332 |
|  |  | 3 | -768.142 | 1580.284 | 1653.71 | 1584.003 | 0.953 | 119.321 | 0.097 |
|  |  | 4 | -738.342 | 1532.683 | 1626.134 | 1537.417 | 0.822 | 57.796 | 0.109 |
|  |  | 5 | -711.163 | 1490.326 | 1603.802 | 1496.074 | 0.864 | 52.711 | 0.164 |
| Mothers | T1 | 2 | -1212.11 | 2456.222 | 2515.268 | 2464.527 | 0.894 | 192.839 | 0.094 |
|  |  | 3 | -1161.25 | 2366.493 | 2447.681 | 2377.912 | 0.744 | 98.834 | 0.115 |
|  |  | 4 | -1132.41 | 2320.81 | 2424.14 | 2335.343 | 0.784 | 56.042 | 0.324 |
|  |  | 5 | -1114.45 | 2296.892 | 2422.364 | 2314.539 | 0.809 | 34.896 | 0.390 |
|  | T3 | 2 | -918.916 | 1869.832 | 1925.048 | 1874.336 | 0.972 | 138.009 | 0.291 |
|  |  | 3 | -869.005 | 1782.01 | 1857.933 | 1788.204 | 0.777 | 96.860 | 0.154 |
|  |  | 4 | -836.841 | 1729.681 | 1826.311 | 1737.564 | 0.815 | 62.420 | 0.171 |
|  |  | 5 | -821.226 | 1710.452 | 1827.787 | 1720.024 | 0.827 | 30.303 | 0.676 |

*Note.* LL = log likelihood; AIC = Akaike information criterion; BIC = Bayesian information criterion;

aBIC = adjusted BIC; LMR-LRT = Lo-Mendell-Rubin likelihood ratio test.

Supplemental Table 3. Latent Class Transitions from Time 1 to Time 3

| **Fathers**  Baseline (T1) | | 1-year Follow-up (T3) | | | Total |
| --- | --- | --- | --- | --- | --- |
|  |  | *High positive* (%) | *Moderate positive* (%) | *Coercive* (%) |  |
|  | *High positive* | 104 (87) | 14 (12) | 1 (1) | 119 |
|  | *Moderate positive* | 101 (69) | 36 (25) | 9 (6) | 146 |
|  | *Coercive* | 12 (52) | 9 (39) | 2 (9) | 23 |
| Total | | 217 | 59 | 12 |  |
| **Mothers**  Baseline (T1) | | 1-year Follow-up (T3) | | | Total |
|  |  | *High positive* (%) | *Moderate positive* (%) | *Coercive* (%) |  |
|  | *High positive* | 66 (81) | 15 (19) | 0 (0) | 81 |
|  | *Moderate positive* | 112 (58) | 71 (37) | 9 (5) | 192 |
|  | *Coercive* | 21 (62) | 11 (32) | 2 (6) | 34 |
| Total | | 199 | 97 | 11 |  |
